# Very Early‐Stage Detection Is Associated With Improved Survival in Patients With Unifocal Hepatocellular Carcinoma

**DOI:** 10.1111/apt.70438

**Published:** 2025-10-31

**Authors:** Thomas Hunold, Karim Seif El Dahan, Suraj Pai, Amit G. Singal, Neehar D. Parikh

**Affiliations:** ^1^ Division of Gastroenterology and Hepatology University of Michigan Ann Arbor Michigan USA; ^2^ Division of Digestive and Liver Diseases University of Texas Southwestern Medical Center Dallas Texas USA

**Keywords:** hepatocellular carcinoma, survival, T1a stage, T1b stage

## Abstract

**Background and Aims:**

The goal of hepatocellular carcinoma (HCC) surveillance is to improve early HCC detection; however, the incremental benefits of detection of T1a tumours compared to T1b tumours are unclear. We aimed to evaluate the survival of patients with HCC detected at a T1a stage compared to T1b stage.

**Methods:**

We conducted a multicentre retrospective study of adult patients from three sites in the United States who were newly diagnosed with unifocal HCC (based on LIRADS v2018), measuring between 1.0 and 3.0 cm at diagnosis between July 2013 and November 2022. All patients were required to have Child‐Turcotte‐Pugh (CP) class A or B cirrhosis and undergo timely treatment within 90 days of diagnosis. Multivariable Cox proportional hazard models were used to evaluate associations between tumour size (T1a vs. T1b) and overall survival and transplant‐free survival.

**Results:**

Of 140 eligible patients (median age 67 years, 72% male), 88 had T1a and 52 had T1b HCC. Median overall survival was 5.5 (95% CI: 3.79–NE (Not Evaluable)) years for patients with T1a HCC versus 3.1 (95% CI: 2.52–4.63) years for those with T1b HCC (*p* = 0.019). In multivariable analysis, mortality was significantly associated with Child Pugh B (vs. Child Pugh A; HR: 2.362; 95% CI: 1.39–4.93), higher logarithm transformed AFP (HR: 1.42; 95% CI: 1.20–1.69) and lesion size in cm (HR: 2.95; 95% CI: 1.69–5.16).

**Conclusions:**

Detection of HCC at a T1a stage conveys a survival advantage compared to T1b stage, underscoring a continued need for improvements in HCC surveillance efficacy.

## Graphical abstract

In a multicentre cohort of patients with unifocal HCC, patients with very early stage disease had a survival advantage over patients diagnosed at an early stage. Factors associated with mortality included worse liver function, larger tumour size and higher AFP levels.

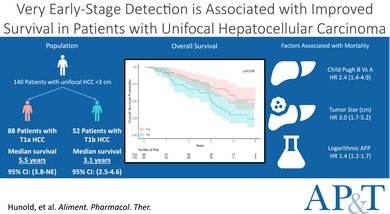

**Abbreviations**
AFP: alpha fetoprotein
CP: Child‐Pugh class
HBV: hepatitis B virus
HCC: Hepatocellular carcinoma
HCV: hepatitis C virus
MASLD: metabolic dysfunction‐associated steatotic liver disease
MELD: model for end‐stage liver disease
SVR: sustained viral response

## 1 Introduction

Hepatocellular carcinoma (HCC) is the third leading cause of cancer‐related death worldwide and the sixth most commonly diagnosed cancer [1, 2]. HCC is a unique malignancy, as it can be diagnosed both by tissue biopsy and non‐invasively through imaging characteristics on abdominal contrast‐enhanced CT or MRI [3]. Current guidelines recommend semi‐annual HCC surveillance using abdominal ultrasound and serum alpha fetoprotein (AFP) in at‐risk patients [4, 5, 6].

HCC diagnosed through surveillance is associated with diagnosis at an earlier stage and prolonged survival compared to HCC due to symptoms or incidentally [7]. However limitations of current surveillance modalities, including suboptimal sensitivity and poor adherence, have prompted evaluation of alternate surveillance methodologies, such as blood‐based biomarkers or MRI/abbreviated MRI based surveillance [8, 9, 10, 11, 12]. The PRIUS study, for example, evaluated MRI versus ultrasound in at‐risk patients and showed superiority of MRI for early detection; however, most of the HCCs were < 2 cm in size [9]. Similarly, recent data evaluating a blood‐based biomarker methylated DNA panel for HCC surveillance, showed superiority of the panel over ultrasound in a cross‐sectional design; however, the sensitivity of ultrasound was 28% with the biggest difference in sensitivity between the blood‐based biomarker and ultrasound being reported for lesions < 2 cm in size [10].

There remains controversy surrounding the clinical implications and optimal management of T1a tumours (unifocal, < 2 cm in size) compared to T1b tumours (unifocal, 2–3 cm in size) [13]. The size of small, unifocal lesions has unclear implications as both are amenable to curable therapies. Further, in the United States, to qualify for model for end‐stage liver disease (MELD) exception points in liver transplant listing, a patient must have at least stage T1b disease (unifocal tumour ≥ 2 cm), with smaller tumours (T1a HCC) not qualifying for MELD exceptions [14]. For patients with T1a tumours, many centres will employ a wait and not ablate strategy to allow for patients to progress to T1b disease [15]. Therefore, understanding differences in prognosis for T1a compared to small T1b HCC has important implications for both clinical trials evaluating emerging surveillance methods as well as clinical management of these patients in practice. Herein, we compared clinical outcomes between patients with T1a HCC and those with unifocal small T1b HCC in a multi‐centre cohort of patients from the United States.

## 2 Methods

### 2.1 Design

We conducted a multicentre, retrospective study including three health systems in the North American Liver Cancer Consortium: University of Michigan, UT Southwestern Medical Center and Parkland Health [16, 17, 18]. Our cohort included adult patients (> 18 years old) with unifocal HCC, based on LIRADS v2018 or histologic diagnosis, measuring between 1.0 and 3.0 cm at diagnosis, diagnosed between July 2013 and November 2022 [3]. Patients with active non‐HCC malignancy, Child‐Pugh C cirrhosis or multifocal disease were excluded. For the primary analysis, we only included patients who underwent timely treatment within 90 days of diagnosis to exclude patients being monitored to obtain MELD exception points prior to transplantation [19]. Prior studies are conflicted on whether therapeutic delays of > 90 days impact survival [19, 20, 21, 22, 23, 24, 25]. We conducted a secondary analysis comparing all patients with T1a HCC (including those with delayed treatment) and those with T1b disease. All sites had institutional review board approval for the conduct of the study and data were shared between sites using institutional data use agreements.

### 2.2 Data Collection

Patients were identified by querying existing databases of patients with newly diagnosed HCC at each site. A REDCap database was shared to collect additional data elements unique to this study. We collected patient demographics, liver disease aetiology and severity, HCC tumour burden, HCC‐directed treatments and clinical outcomes including dates of death or liver transplantation.

Liver disease aetiology was classified, in a hierarchical manner, as follows: HCV post‐sustained viral response (SVR), viremic hepatitis C virus (HCV), hepatitis B virus (HBV), alcohol‐related cirrhosis, other, metabolic dysfunction‐associated steatotic liver disease (MASLD) or cryptogenic. Tumour burden and responses to treatment were determined via imaging reports or multidisciplinary tumour board reports.

### 2.3 Primary/Secondary Outcomes

The primary outcome of interest was overall survival from the time of HCC diagnosis, stratified by tumour size (1.0–2 cm [T1a] vs. > 2.0–3.0 cm [T1b]) for patients treated within 90 days of diagnosis. Prespecified secondary analyses included transplant‐free survival and progression‐free survival.

### 2.4 Statistical Analysis

Descriptive statistics were calculated for the overall cohort, as well as stratified by T1a versus T1b stage. We performed univariable analysis using chi‐square and Student's *t*‐tests/Mann–Whitney tests for categorical and continuous variables, respectively. Kaplan–Meier and Cox proportional hazards models were constructed for survival times, with patients censored on December 31st, 2023, if they had not undergone liver transplant or died earlier. Variables included in Cox proportional hazards models were determined based on clinical relevance. Subgroup exploratory analyses were completed to evaluate outcomes by liver disease aetiology (viral vs. non‐viral), Child Pugh class, initial treatment type, comparing all T1a tumours to all T1b tumours and tumour size by 0.5 cm. A 90‐day landmark analysis was also done in a restricted subgroup of patients who survived to 90 days. Patients who died, had HCC progression or their last clinic visit before this landmark were excluded. These restrictions avoid immortal time bias by synchronizing the start of follow‐up for treated and untreated patients [26, 27]. All analyses were conducted using R using the survival package (R version 4.4.1).

## 3 Results

### 3.1 Patient Characteristics

Of 326 patients with unifocal HCC between 1 and 3 cm in maximum diameter, 140 met inclusion criteria. Reasons for exclusion included delayed treatment (*n* = 131), Child Pugh C cirrhosis (*n* = 44), lack of histologic diagnosis prior to LIRADs guidelines (*n* = 10) and lack of any follow‐up after diagnosis (*n* = 1). Demographic and clinical characteristics of patients, stratified by T1a versus T1b stage, are shown in Table 1. Median age of patients was 67 years (IQR: 12), 72% were male, 81% were Caucasian. The most common liver disease aetiologies were MASLD (29%), Hepatitis C post‐SVR (25%), alcohol‐related cirrhosis (23%) and Hepatitis C pre‐SVR (11%); most patients had Child‐Pugh class A cirrhosis (74%). The diagnosis of HCC was most commonly made by MRI (42%) followed by US (35%) and 59% of patients were diagnosed on a screening test. Thirty percent of patients had pathology confirming the diagnosis. These proportions remained consistent across the T1a and T1b stages. Median maximum diameter of HCC was 1.9 cm (IQR: 0.8), with 88 having T1a HCC and 52 T1b HCC. Patients with T1a and those with T1b HCC had similar median age (67 vs. 67 years), sex distribution (72% vs. 73% male), race/ethnicity (84% vs. 77% Caucasian), liver disease aetiologies (37.8% vs. 32% viral aetiology) and Child‐Pugh distribution (72% vs. 77% Child Pugh class A).

**TABLE 1:** Demographics by tumour size.

	Overall *N* = 140	Stage	
		T1a HCC *N* = 88	T1b HCC *N* = 52
Age in years, Median (Q1–Q3)	67 (61–73)	67 (60–72)	67 (61–74)
Sex, % (*n*)			
Male	72% (101)	72% (63)	73% (38)
Female	28% (39)	28% (25)	27% (14)
Race/Ethnicity, % (*n*)			
Caucasian	81% (114)	84% (74)	77% (40)
Black or African American	10% (14)	5.7% (5)	17% (9)
Hispanic	2.9% (4)	3.4% (3)	1.9% (1)
Asian	2.9% (4)	3.4% (3)	1.9% (1)
American Indian	0.7% (1)	0% (0)	1.9% (1)
Unknown or not reported	2.1% (3)	3.4% (3)	0% (0)
Aetiology of liver disease, % (*n*)			
Alcohol	23% (32)	27% (24)	37% (19)
MASLD	29% (40)	24% (21)	15% (8)
HCV (pre‐SVR)	11% (15)	6.8% (6)	17% (9)
HCV (post‐SVR)	25% (35)	31% (27)	15% (8)
HBV	0% (0)	0% (0)	0% (0)
Cryptogenic	5.7% (8)	3.4% (3)	9.6% (5)
Other	7.1% (10)	8.0% (7)	5.8% (3)
Modality of diagnosis			
US	35% (49)	34% (30)	37% (19)
CT	14% (20)	11% (10)	19% (10)
MRI	42% (59%)	44% (39)	38% (20)
Blood based test	8.6% (12)	10% (9)	5.8% (3)
Screening test			
Yes	59% (82)	63% (55)	52% (27)
No	41% (58)	38% (33)	48% (25)
Pathology			
Yes	30% (42)	27% (24)	35% (18)
No	70% (98)	73% (64)	65% (34)
Laboratory values at diagnosis			
Albumin, Median (Q1–Q3)	3.80 (3.40–4.30)	3.80 (3.50–4.30)	3.70 (3.30–4.30)
AST, Median (Q1–Q3)	47 (33–66)	42 (32–58)	53 (36–77)
ALT, Median (Q1–Q3)	32 (25–46)	31 (23–42)	34 (27–62)
ALP, Median (Q1–Q3)	112 (82–158)	107 (79–142)	126 (102–185)
INR, Median (Q1–Q3)	1.10 (1.09–1.20)	1.10 (1.10–1.25)	1.10 (1.00–1.20)
Platelet count, Median (Q1–Q3)	113 (79–163)	112 (82–160)	116 (72–167)
AFP, Median (Q1–Q3)	5 (3–14)	5 (3–14)	4 (2–14)
MELD‐Na, Median (Q1–Q3)	11.0 (8.0–14.0)	11.0 (8.5–14.0)	10.5 (8.0–13.0)
Child‐Turcotte‐Pugh class, % (*n*)			
A	74% (103)	72% (63)	77% (40)
B	26% (37)	28% (25)	23% (12)
HCC characteristics			
Tumour size at diagnosis, Median (Q1–Q3)	1.90 (1.50–2.30)	1.60 (1.30–1.80)	2.50 (2.25–2.70)
Days to first treatment, Median (Q1–Q3)	52 (39–67)	52 (40–64)	52 (39–71)
First treatment, % (*n*)			
LT	0.7% (1)	1.1% (1)	0% (0)
Resection	7.9% (11)	5.7% (5)	12% (6)
MWA/RFA	51% (72)	55% (48)	46% (24)
TACE	1.4% (2)	0% (0)	3.8% (2)
TARE/Y‐90	3.6% (5)	1.1% (1)	7.7% (4)
SBRT	34% (47)	36% (32)	29% (15)
Systemic Therapy	0% (0)	0% (0)	0% (0)
Clinical trial	0% (0)	0% (0)	0% (0)
Other	1.4% (2)	1.1% (1)	1.9% (1)
None	0% (0)	0% (0)	0% (0)
Outcomes			
Binary outcome, % (*n*)			
Alive	63% (88)	73% (64)	46% (24)
Dead	37% (52)	27% (24)	54% (28)
Outcome, % (*n*)			
LT	10% (14)	10% (9)	9.6% (5)
Undergoing LT evaluation	2.1% (3)	1.1% (1)	3.8% (2)
Wait‐list dropout	0% (0)	0% (0)	0% (0)
Death of patient	37% (52)	27% (24)	54% (28)
Lost to follow‐up	6.4% (9)	5.7% (5)	7.7% (4)
No LT for other reasons	44% (62)	56% (49)	25% (13)

Abbreviations: AFP, alpha‐fetoprotein; ALP, alkaline phosphatase; ALT, alanine aminotransferase; AST, aspartate transaminase; HBV, hepatitis B virus; HCV, hepatitis C virus; INR, international normalised ratio; LT, liver transplant; MASLD, metabolic dysfunction‐associated steatotic liver disease; MELD‐Na, model for end stage liver disease—sodium; MWA, microwave ablation; RFA, radiofrequency ablation; SBRT, stereotactic body therapy; SVR, sustained virologic response; TACE, transarterial chemoembolisation; TARE, transarterial radioembolisation; Y‐90, Yttrium 90.

Over a median follow‐up of 2.2 years (IQR: 2.6 years) and median potential follow‐up using the reverse Kaplan Meier estimator of 3.2 years (IQR: 2.91 years), 37% of patients died, 10% underwent liver transplant and 6.4% were lost to follow‐up. The median number of days to first HCC treatment was 52 days (IQR: 28). The most common treatments were local ablation (51%), stereotactic body radiation therapy (34%) or surgical resection (8%). Treatment allocation was similar between T1a and T1b tumours (*p* = 0.072). Most patients had an objective response (partial or complete) to the first HCC‐directed treatment, including 84% of patients with T1a HCC and 74% of those with T1b HCC (*p* = 0.3). Sixty‐five percent (53 patients) with T1a tumours who were determined to have a complete response compared to 53% (25 patients) with T1b tumours. Of these 38% (20 patients) of patients with T1a tumours experienced recurrence of their disease compared to 56% (14 patients) of patients with T1b tumours.

### 3.2 Overall Survival

**FIGURE 1:**
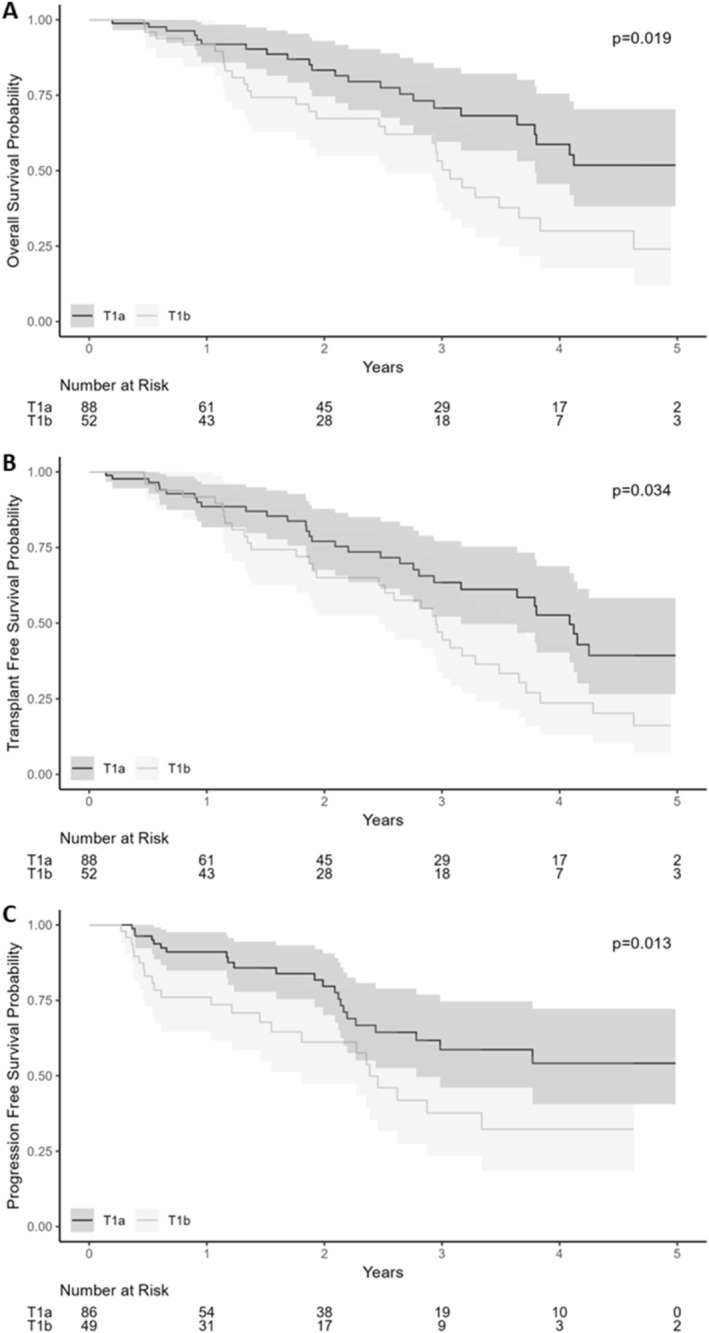
Kaplan Meier Curves for T1a versus T1b HCC treated within 90 days. (A) Overall survival. (B) Transplant free survival. (C) Progression free survival.

Patients with T1a HCC had a median survival of 5.5 years compared to 3.0 years for those with T1b HCC (*p* = 0.019; Figure 1A). Landmark analysis demonstrated 1‐, 3‐ and 5‐year survival of 91.9%, 70.7% and 51.8% for T1a HCC, compared to 91.7%, 53.2% and 24.0% for those with T1b HCC (*p* = 0.002; Figure S1). In multivariable Cox‐proportional hazards analysis, overall survival was associated with age in years (HR: 1.03; 95% CI: 1.00–1.07), aetiology of liver disease, Child‐Pugh class B cirrhosis (HR: 2.62; 95% CI: 1.39–4.93), higher natural logarithm transformed AFP (HR: 1.42; 95% CI: 1.20–1.69) and increasing tumour size in centimetres (HR: 2.95; 95% CI: 1.69–5.16) (Table 2).

**TABLE 2:** Overall survival cox proportional hazards in patients with unifocal early stage hepatocellular carcinoma.

	HR	95% CI	*p*
Age in years	1.03	1.00, 1.07	0.047
Gender			
Male	—	—	
Female	1.58	0.88, 2.86	0.13
Liver disease aetiology			
MASH/MASLD	—	—	
EtOH	1.67	0.74, 3.77	0.2
HCV (pre‐SVR)	0.75	0.32, 1.78	0.5
HCV (post‐SVR)	0.37	0.12, 1.16	0.089
HBV	0.00	0.00, Inf	> 0.9
Cryptogenic	1.57	0.45, 5.53	0.5
Other	2.95	0.98, 8.83	0.053
Child‐Pugh classification			
A	—	—	
B	2.62	1.39, 4.93	0.003
Log(AFP) at diagnosis	1.42	1.20, 1.69	< 0.001
Lesion size	2.95	1.69, 5.16	< 0.001

Abbreviation: Log(AFP), natural log of AFP.

### 3.3 Subgroup Analyses for Overall Survival

Results were consistent in subgroups of patients with viral and non‐viral aetiologies of cirrhosis, although the difference failed to reach statistical significance in the non‐viral subgroup. Median survival for patients with T1a and T1b HCC amongt those with viral liver disease, was 6.05 (5.51–NE) and 4.63 (2.95–NE) years, respectively (*p* = 0.12; Figure S2). In those with non‐viral aetiologies, median survival for patients with T1a HCC was 3.64 years (2.76–NE) compared to 2.96 years (1.87–NE) for those with T1b HCC (*p* = 0.085; Figure S3).

When analysed by Child‐Pugh class (CP), patients with CP A cirrhosis had a longer median survival of 4.63 years (3.64–NE) compared to patients with CP B cirrhosis of 2.96 years (1.87–4.12) (*p* = 0.004; Figure S4). Among patients with CP A cirrhosis, those with T1a disease had a significantly longer overall survival at 5 years of 65.2% (49.9%–85.2%) compared to 27.6% (13.5%–56.5%) for T1b disease (*p* = 0.009; Figure S5). The median survival was not evaluable in the T1a group. While among patients with CP B cirrhosis, the difference in survival was not statistically significant, the trend towards longer overall survival for T1a patients (median 3.79; 95%: CI: 1.87–NE) compared to T1b patients (median 2.45; 95% CI: 1.32–NE) (*p* = 0.3; Figure S6).

When evaluated by the most common treatment types (surgical resection, MWA/RFA, SBRT), there was a no difference in survival (*p* = 0.13; Figure S7). Patients who underwent surgical resection (*n* = 11) had a median survival that was not evaluable compared to those who underwent MWA/RFA or SBRT of 4.12 years (3.49–NE) and 3.07 years (2.52–NE) respectively.

We conducted an exploratory analysis comparing further granularity in size strata (1.0–1.4 cm [*n* = 29] vs. 1.5–1.9 cm [*n* = 55] vs. 2.0–2.4 cm [*n* = 37] vs. 2.5–3.0 cm [*n* = 29]). Median overall survivals for each group were 5.51, 4.08, 3.49 and 1.93 years, respectively (*p* < 0.001; Figure S8).

We also evaluated all patients with T1a and T1b disease regardless of whether they received therapy for HCC. The trend towards longer overall survival for patients with T1a (median: 4.33; 95% CI: 3.80–NE) compared to patients with T1b (median: 3.66; 95% CI: 3.07–NE) (*p* = 0.4; Figure S9).

### 3.4 Secondary Outcomes: Transplant‐Free and Progression‐Free Survival

Patients with T1a HCC had a median transplant‐free survival of 4.08 years (IQR) compared to 2.95 years for those with T1b HCC (*p* = 0.034; Figure 1B). Landmark analyses demonstrated 1‐, 3‐ and 5‐year survival of 90.5%, 64.3% and 39.1% for T1a HCC, compared to 91.2%, 43.8% and 12.5% for those with T1b HCC (*p* = 0.008; Figure S10). In multivariable analysis, transplant‐free survival was associated with disease aetiology, CP class, logarithmic AFP and the size of the tumour (Table 3).

Patients with T1A HCC had a not evaluable median progression‐free survival compared to 2.38 years for those with T1B HCC (*p* = 0.13; Figure 1C). Progression‐free survival did not significantly differ between patients treated with surgical resection, MWA/RFA and SBRT (*p* = 0.5; Figure S11).

## 4 Discussion

In this multi‐centre, retrospective cohort study, detection of HCC at a T1a stage was associated with significantly improved overall and transplant‐free survival for patients compared to T1b stage HCC. These results were consistent across examined subgroups including liver disease aetiology, Child Pugh class and type of initial HCC treatment. Consistent with these results, prior studies have found that treatment with RFA at a smaller size is associated with improved survival [28]. While prior studies have consistently demonstrated benefits of early detection at Barcelona‐Clinic Liver Cancer Stage 0/A this is the first study in a contemporary cohort to demonstrate T1a stage detection results in improved survival compared to patients with T1b stage HCC to the best of our knowledge. These data have important implications when considering goals of HCC surveillance programmes—both in clinical trials as well as in routine clinical practice (Table 3).

**TABLE 3:** Transplant free survival cox proportional hazards in patients with unifocal early stage hepatocellular carcinoma.

	HR	95% CI	*p*
Age in years	1.02	0.98, 1.05	0.3
Gender			
Male	—	—	
Female	1.24	0.71, 2.18	0.4
Liver disease aetiology			
MASH/MASLD	—	—	
EtOH	1.14	0.56, 2.31	0.7
HCV (pre‐SVR)	0.43	0.19, 0.98	0.046
HCV (post‐SVR)	0.35	0.14, 0.87	0.023
HBV	0.00	0.00, Inf	> 0.9
Cryptogenic	0.90	0.27, 3.05	0.9
Other	1.85	0.65, 5.26	0.2
Child‐Pugh classification			
A	—	—	
B	2.62	1.51, 4.55	< 0.001
Log(AFP) at diagnosis	1.26	1.07, 1.49	0.005
Lesion size	2.36	1.45, 3.83	< 0.001

The survival benefit of T1a HCC detection can be attributed to several factors, including the relationship between tumour size and vascular invasion with decreased efficacy of thermal ablative therapies with increasing tumour size [28]. Recent studies have also found differences in doubling time between small and larger tumours that could favour improved access to curative therapies [29]. Alternatively, there is a possibility of overdiagnosis of non‐HCC‐sized T1a lesions as HCC. The positive predictive value of imaging features for HCC diagnosis is lower with smaller tumour size [30, 31, 32]. Indeed, the positive predictive value of imaging for HCC in T1a HCC underscores the rationale for not awarding MELD exception points at this time [33, 34, 35]. Our results show that despite the low risk of progression, patients treated for their T1a lesions experience superior overall and transplant‐free survival. Given the excellent outcomes of T1a disease, our study supports current transplant exception policy. Additionally, in our exploratory analysis patients with T1a disease who had a treatment delay experience equivalent survival to those who present with T1b disease, suggesting that a wait and not ablate strategy could have negative consequences [35].

Current strategies emphasise the importance of semi‐annual surveillance compared to annual to increase the proportion of tumours diagnosed at early stages [36]. Efforts to increase the uptake of surveillance for HCC and evaluation of novel surveillance methodologies offer opportunities to increase the proportion of patients presenting with T1a HCC [7, 37]. Promising strategies such as blood‐based biomarkers may offer an avenue for the increased uptake of surveillance; however, these lack sufficient sensitivity for T1 disease [10]. Should the goal become the detection of T1 disease, MRI may be the screening test of choice in at‐risk groups [9]. Although our data suggest benefits of the early diagnosis of T1a HCC, the harms of the repeated testing must be balanced with the benefits. Recent evidence collected using a modified Delphi method of 21 content suggested that patients value the benefits more than harms of surviellance [38]. The use of precision approaches to match patient risk of HCC development and surveillance test quality will play a crucial role in this goal to minimise the harms of surveillance despite this. Understanding the most effective way to manage both T1a and T1b patients in the context of an evolving treatment landscape, will become increasingly important. This study reinforces the need to connect patients to multidisciplinary treatment teams to coordinate expedited treatment for even early‐stage HCC.

Other factors associated with improved overall survival included the degree of liver dysfunction, AFP at diagnosis and liver disease aetiology. Specifically, patients with viral aetiologies had prolonged survival compared to those with non‐viral liver disease. These data are consistent with prior studies potentially reflecting lower uptake of HCC surveillance in patients with non‐viral diseases leading to diagnoses at later stages, less access to curative therapies due to increased numbers of comorbidities and more aggressive tumour biology [39, 40, 41, 42].

Our study must be interpreted within several contextual factors. First, this is a retrospective study which inherently predisposes the study to confounding, ascertainment and selection biases. Second, the distribution of aetiologies of liver disease—namely, being HCV‐predominant—in our cohort does not reflect the modern epidemiology of cirrhosis and HCC. Third, we were underpowered to detect differences in subgroup analyses, and the results of this study would benefit from verification in larger cohorts. Fourth, this is a multi‐centre study, with inter‐institutional variability in treatment patterns. We believe the weaknesses of the study are offset by its strengths, including a multicentre cohort of patients with well characterised HCC and clinical course to allow for ascertainment of outcomes. Future studies should continue to investigate the differences in outcomes for patients with small HCC in larger cohorts and aim to understand the treatment benefit of specific therapeutic strategies for early‐stage HCC.

We have shown that patients with T1a HCC have improved survival compared to T1b HCC, underscoring a continued need to optimise the efficacy of surveillance strategies to optimise very early‐stage detection when possible.

## Author Contributions

**Thomas Hunold:** writing – original draft, methodology, formal analysis. **Karim Seif El Dahan:** writing – review and editing, data curation. **Suraj Pai:** conceptualization, writing – original draft, data curation. **Amit G. Singal:** conceptualization, investigation, writing – review and editing, methodology, formal analysis. **Neehar D. Parikh:** formal analysis, supervision, methodology, writing – review and editing, investigation, conceptualization.

## Conflicts of Interest

Amit G. Singal has served as a consultant or on advisory boards for Genentech, AstraZeneca, Eisai, Bayer, Exelixis, Merck, Elevar, Boston Scientific, Sirtex, FujiFilm Medical Sciences, Exact Sciences, Helio Genomics, Roche, Glycotest, Abbott, DELFI, IMCare and Universal Dx. Neehar D. Parikh has served as a consultant or on advisory boards for Exact Sciences, AstraZeneca, Eisai, Exelixis, Sirtex, Fujifilm Medical and Genentech. The other authors declare no conflicts of interest.

## Supporting information


**Figures S1–S11:** apt70438‐sup‐0001‐FigureS1‐S11.docx.

## Data Availability

The data that support the findings of this study are available on request from the corresponding author. The data are not publicly available due to privacy or ethical restrictions.
