# Supplementary material for: Very Early‐Stage Detection Is Associated With Improved Survival in Patients With Unifocal Hepatocellular Carcinoma
Source: Aliment Pharmacol Ther. 2025 Oct 31;63(6):850–8. doi: 10.1111/apt.70438 (PMC12934548; doi:10.1111/apt.70438)

Supplemental tables and figures for Very Early-Stage Detection is Associated with Improved Survival in Patients with Unifocal Hepatocellular Carcinoma.

Supplemental Figure 1. Landmark Analysis for Overall Survival


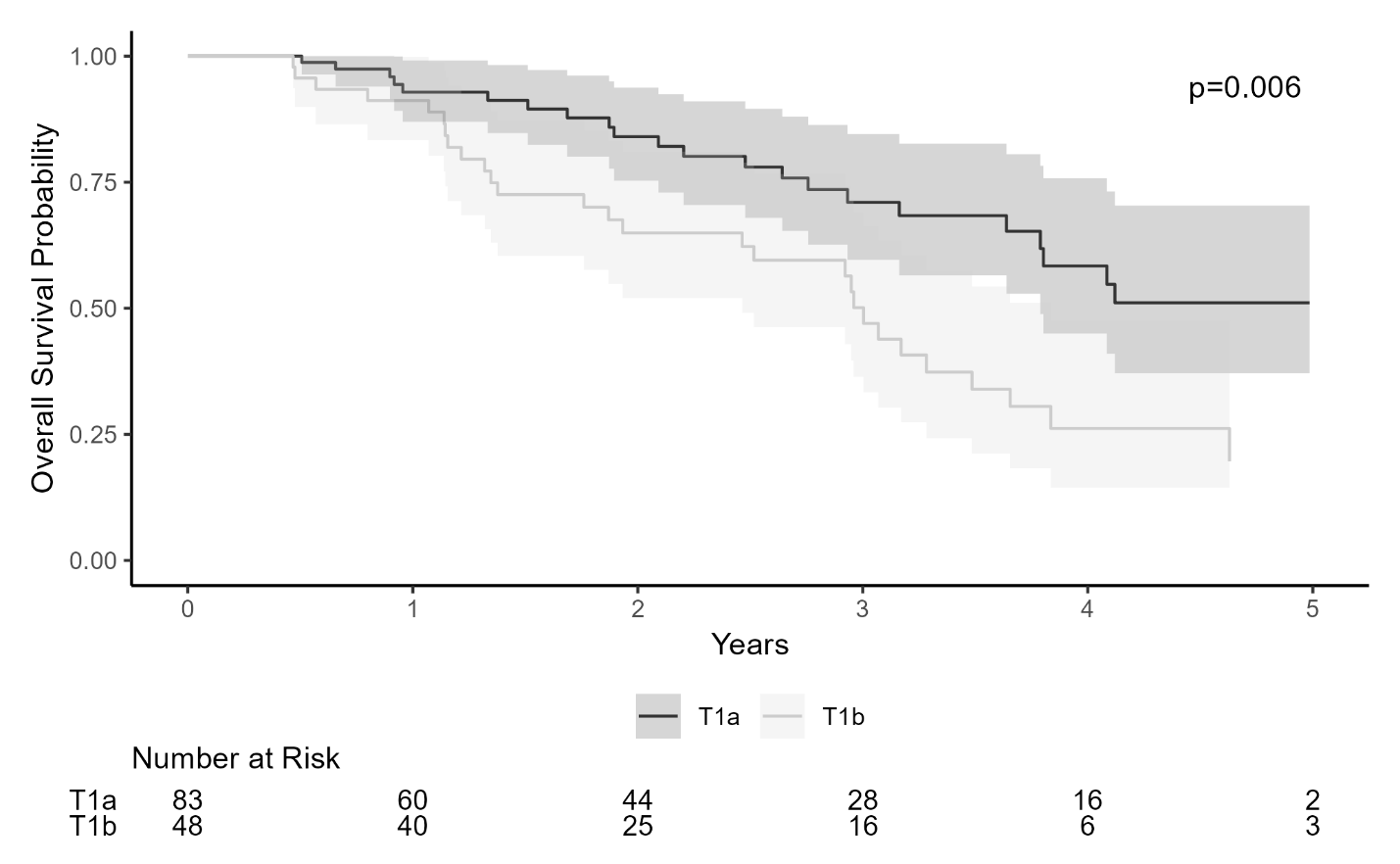


Supplemental Figure 2. Overall Survival for Viral Etiology of Cirrhosis by Tumor Stage


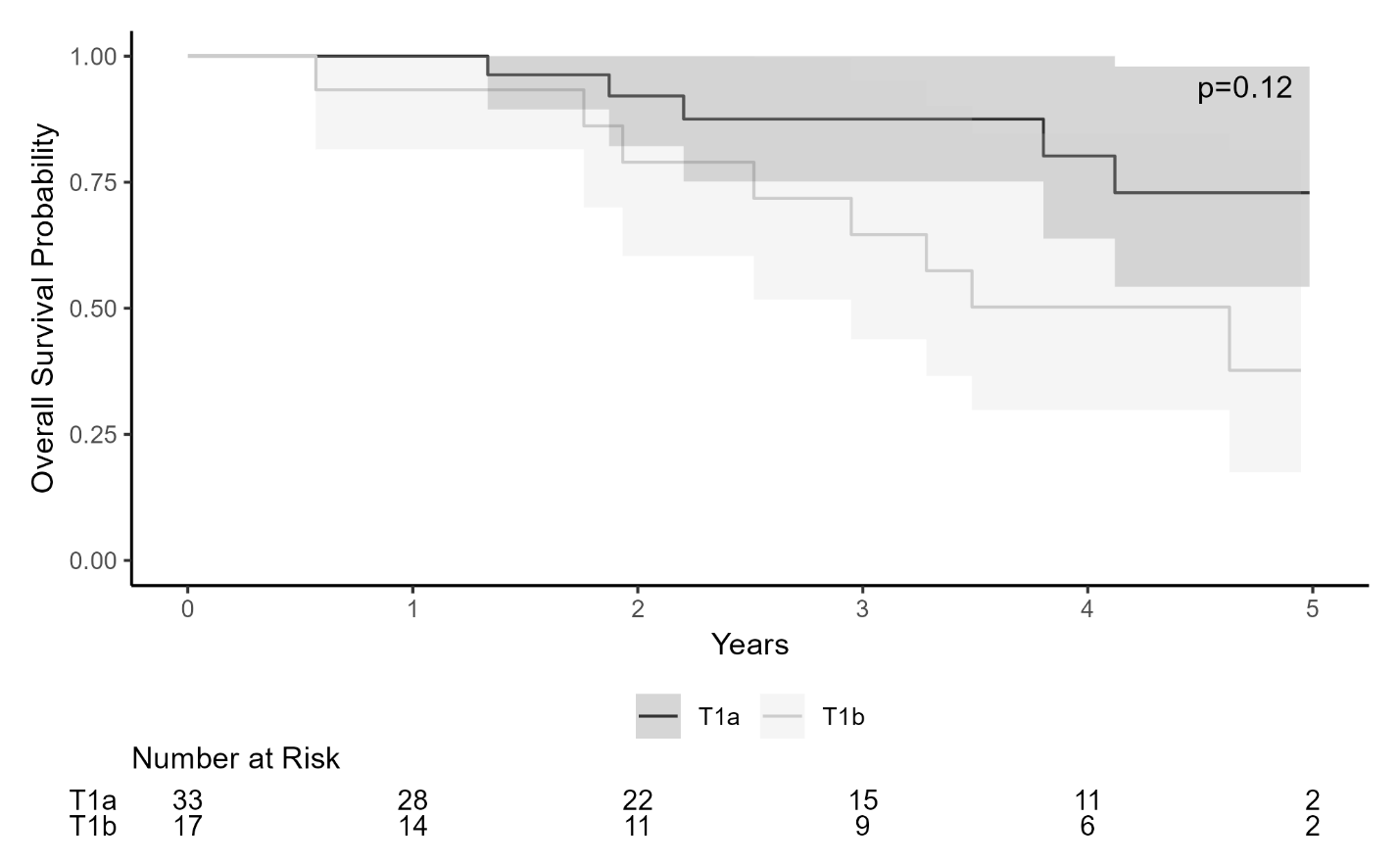


Supplemental Figure 3. Overall Survival for Non-Viral Etiology of Cirrhosis by Tumor Stage


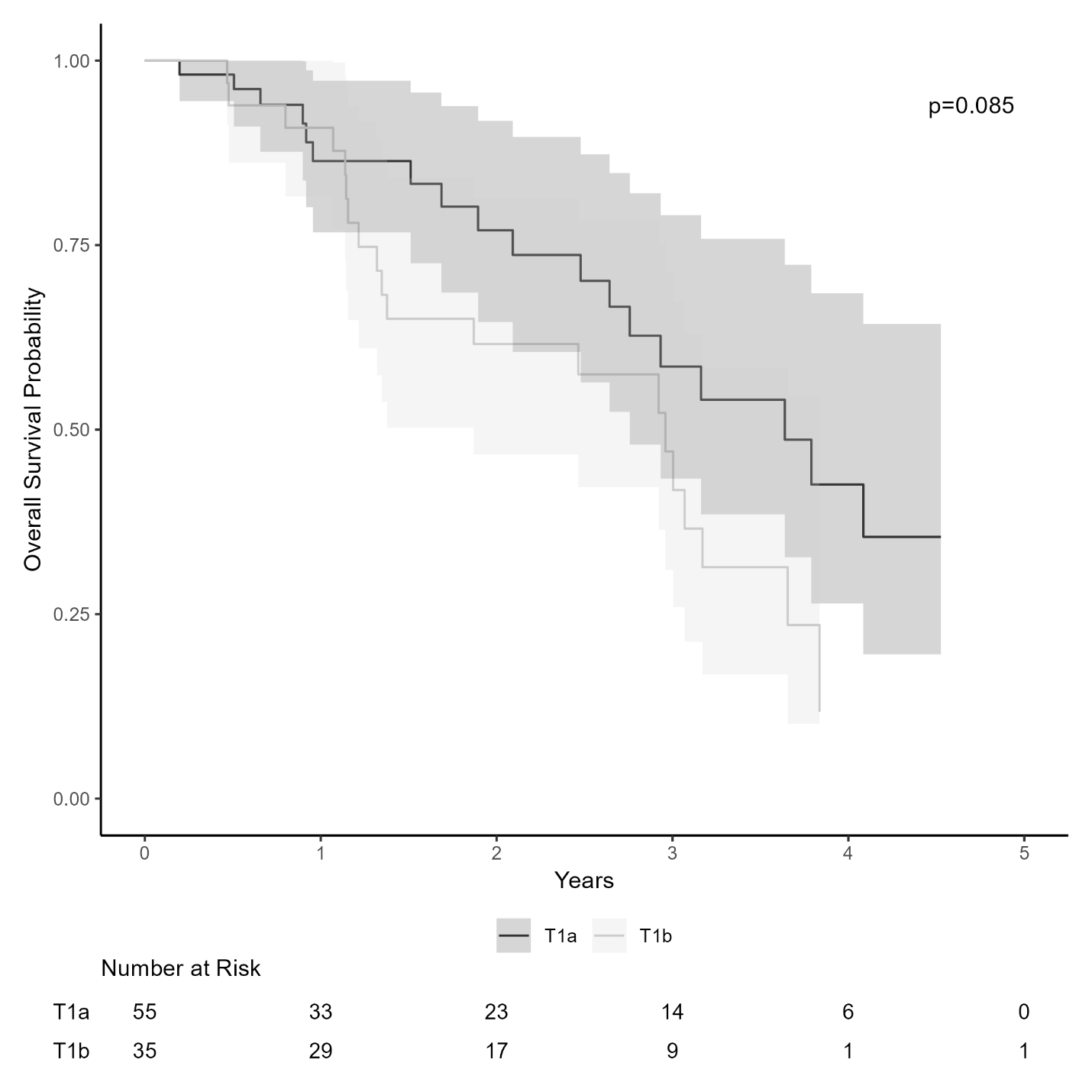


Supplemental Figure 4. Overall Survival by CP class


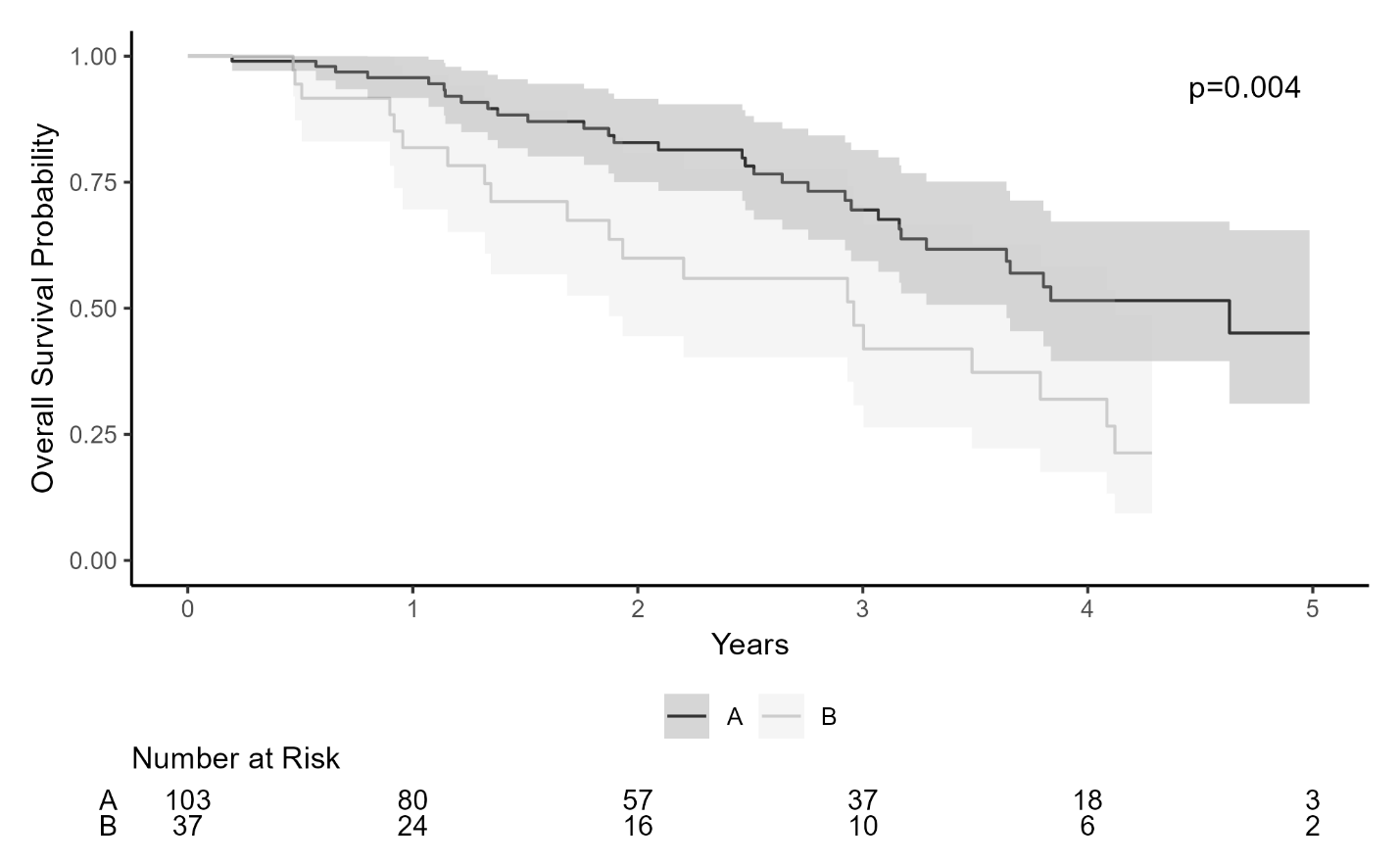


Supplemental Figure 5. Overall Survival by Stage for CP A Cirrhosis


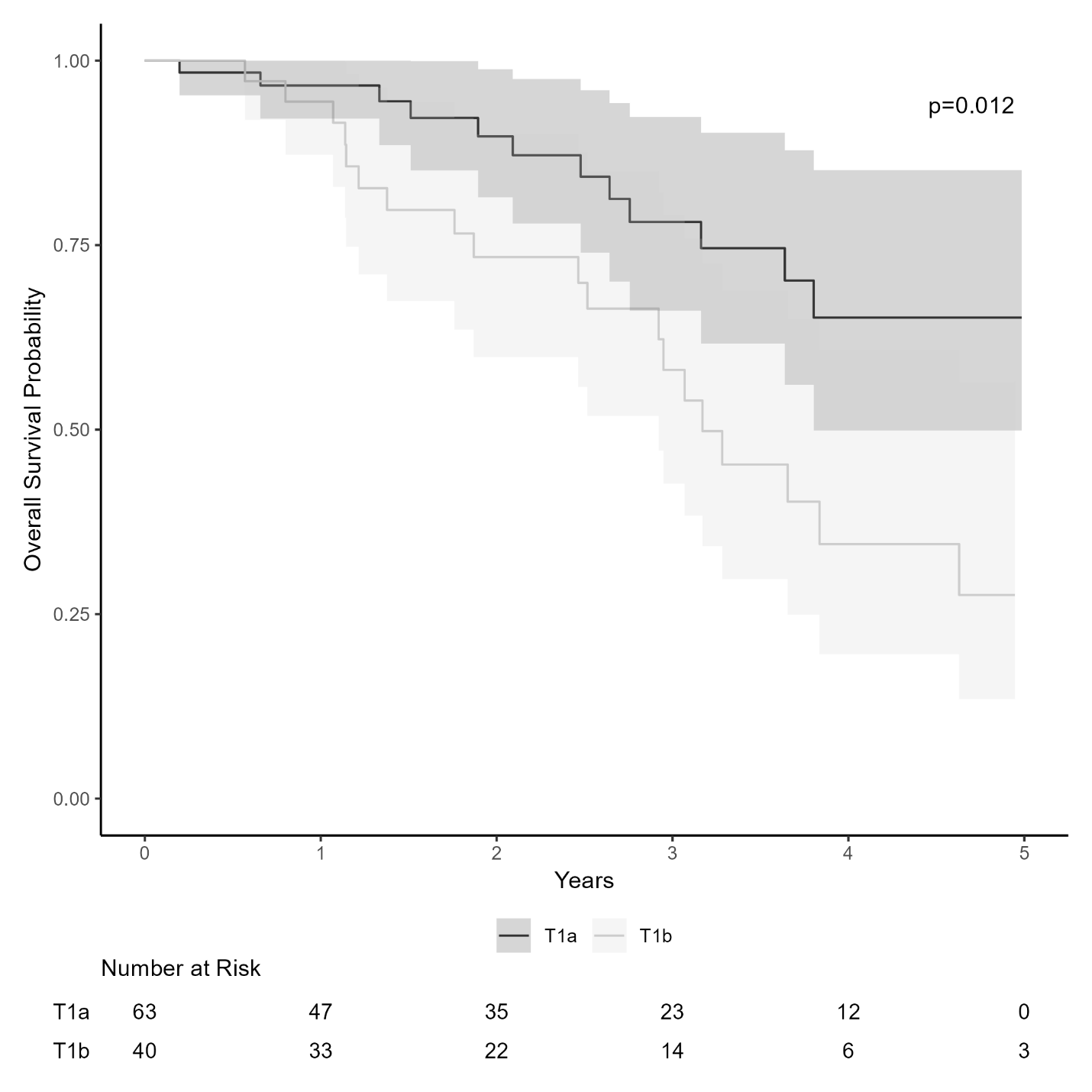


Supplemental Figure 6. Overall Survival by Stage for CP B Cirrhosis


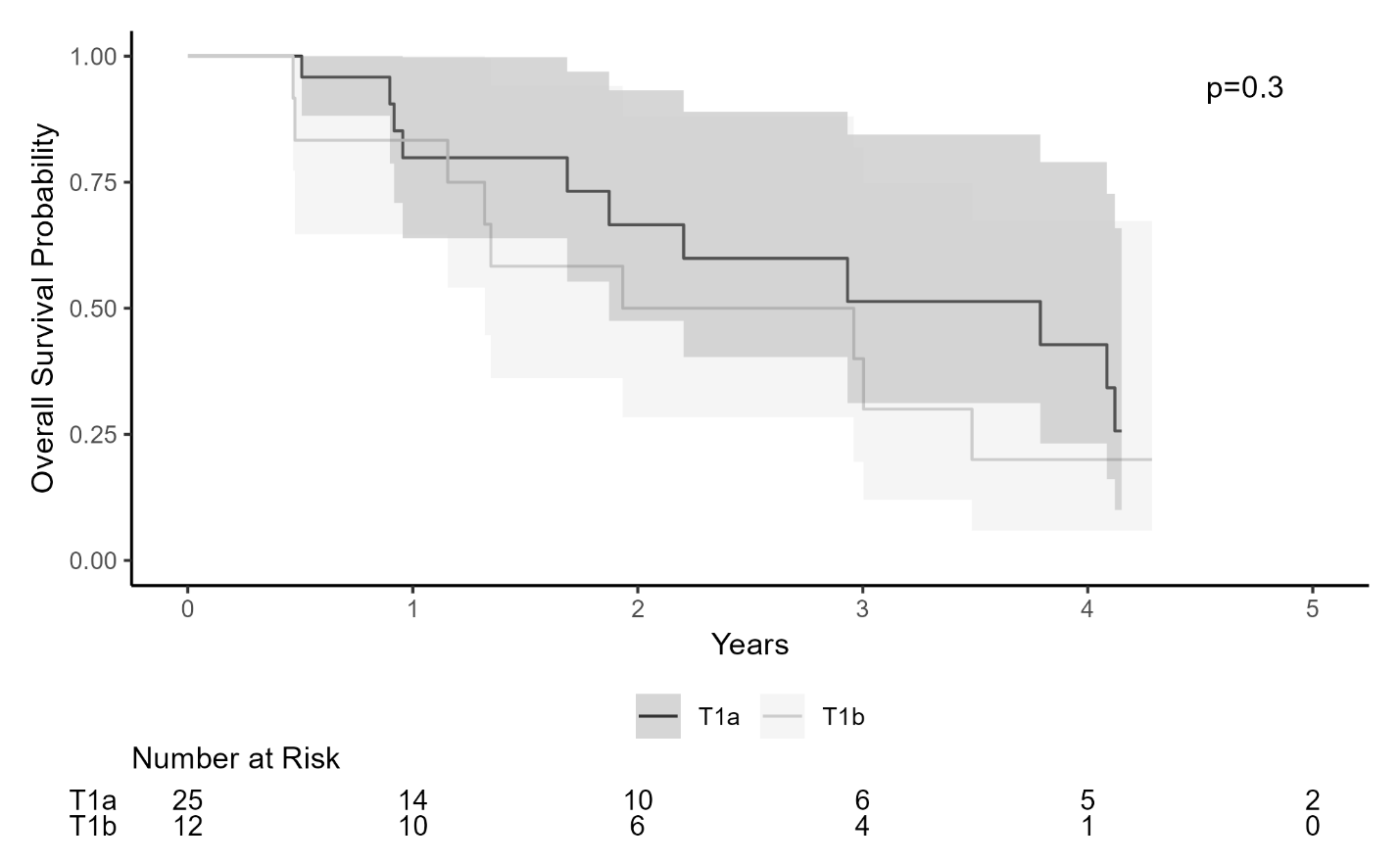


Supplemental Figure 7. Overall Survival by Initial Treatment Type


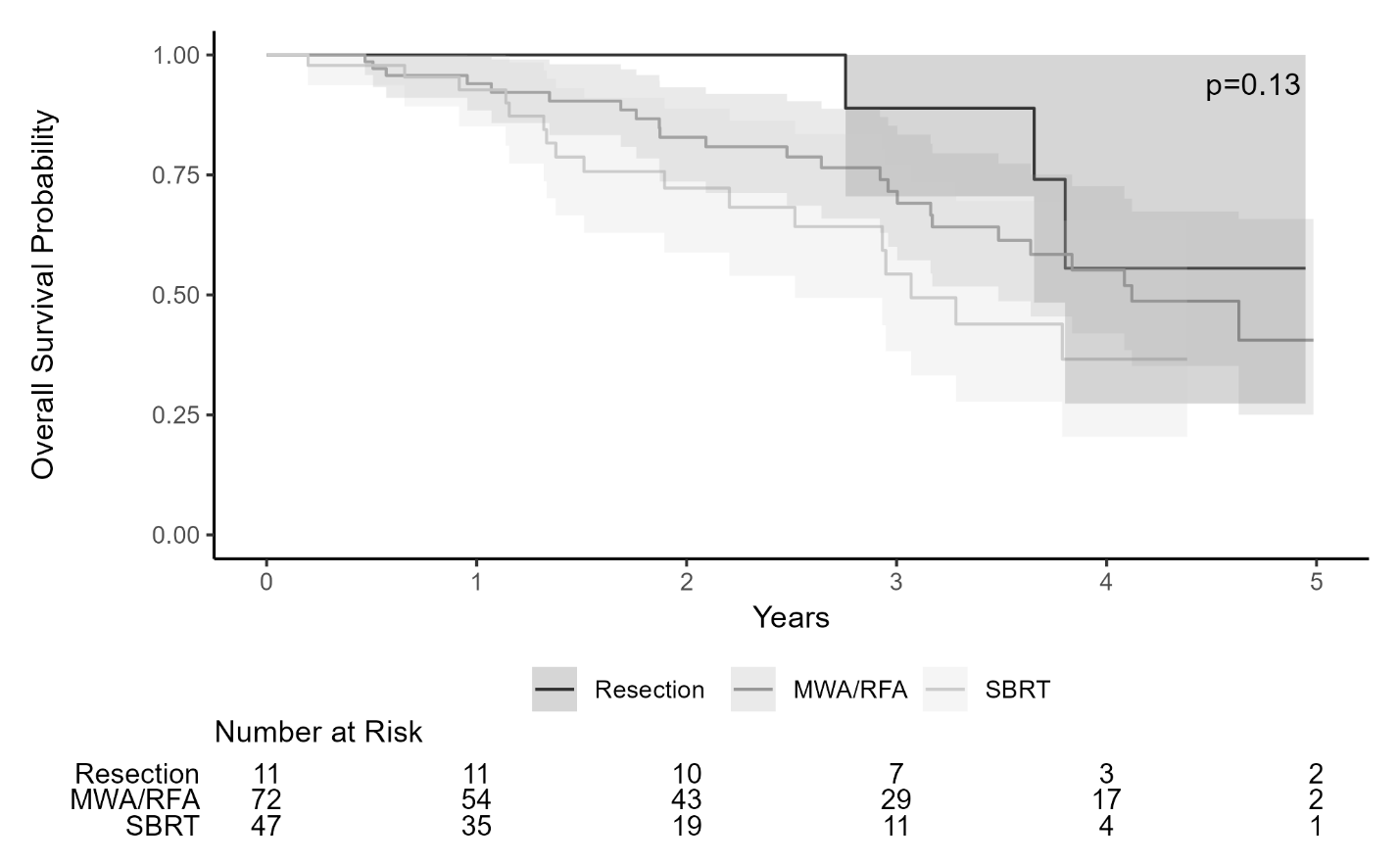


Supplemental Figure 8. Overall Survival by 0.5 cm Tumor size


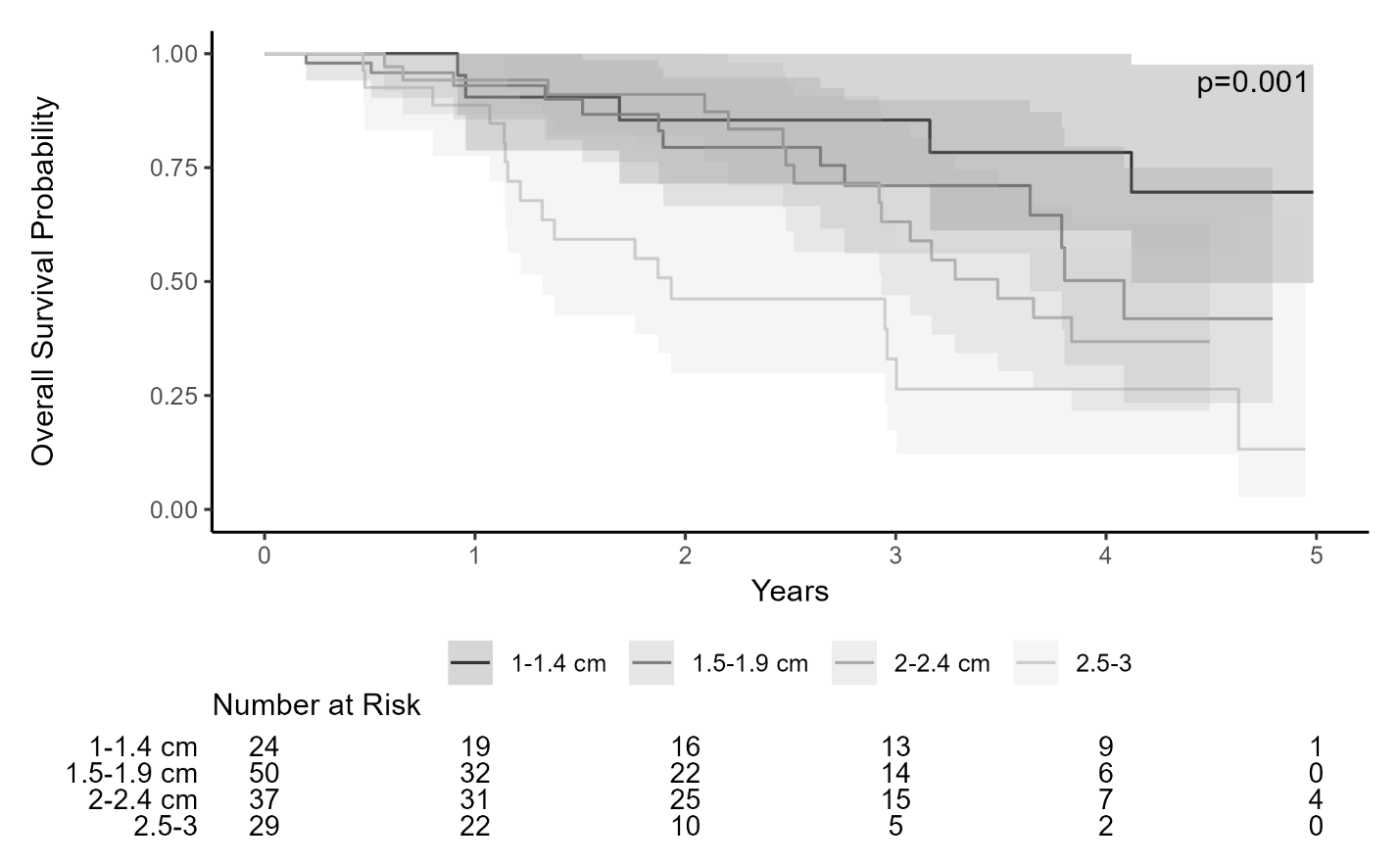


Supplemental Figure 9. Overall Survival for all T1a versus all T1b


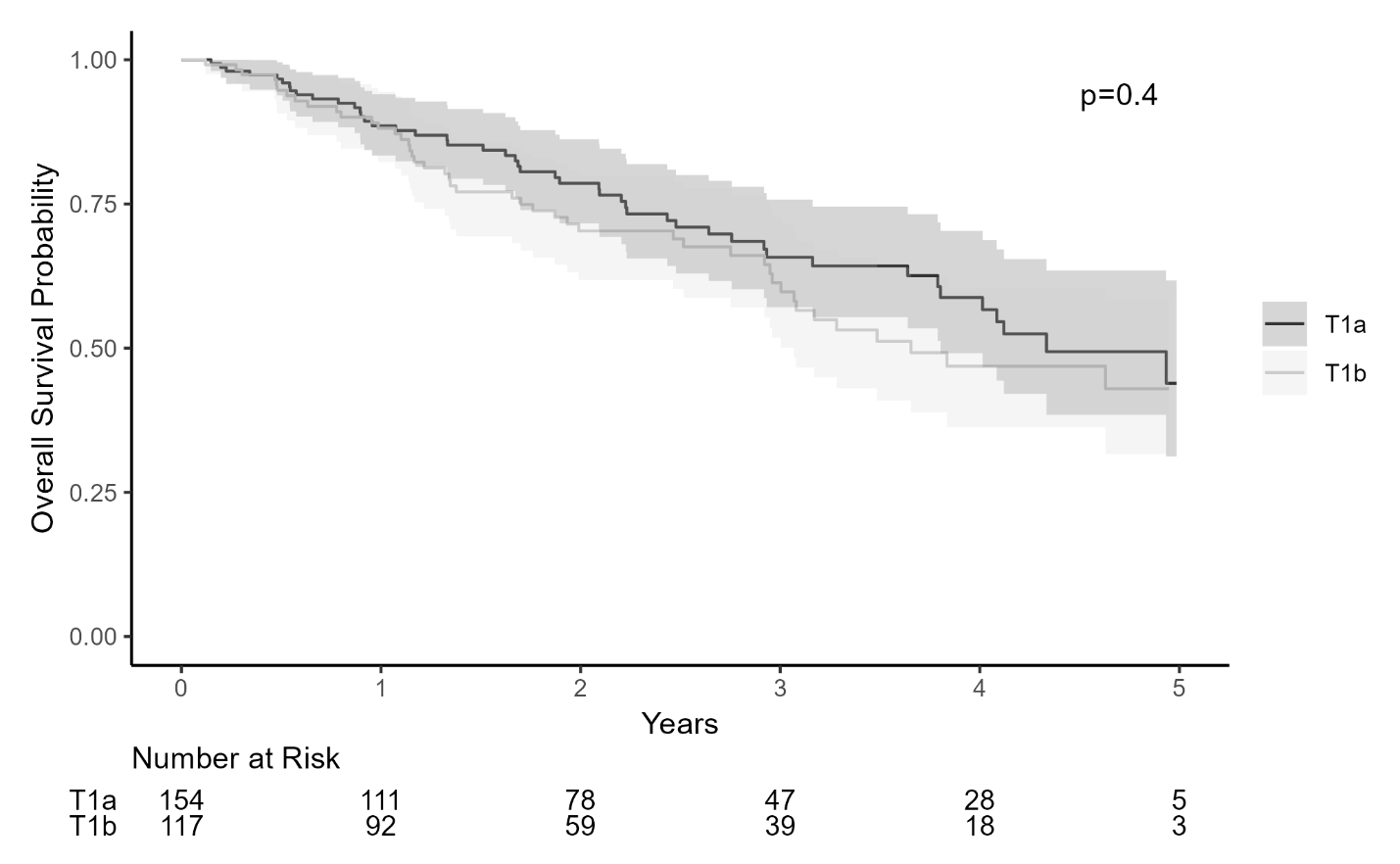


Supplemental Figure 10. Landmark Analysis for Transplant Free Survival


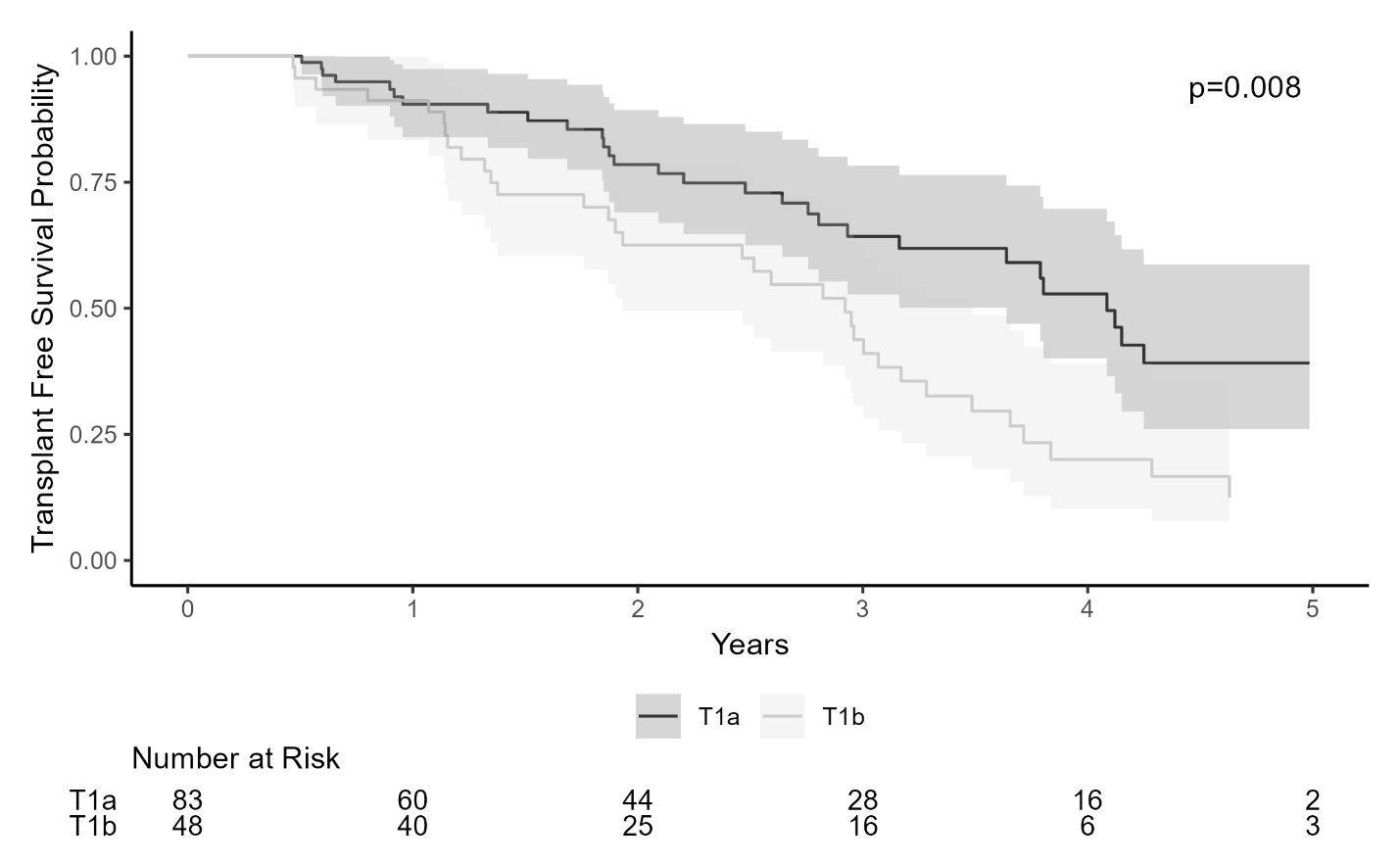


Supplemental Figure 11: Progression Free Survival by first treatment


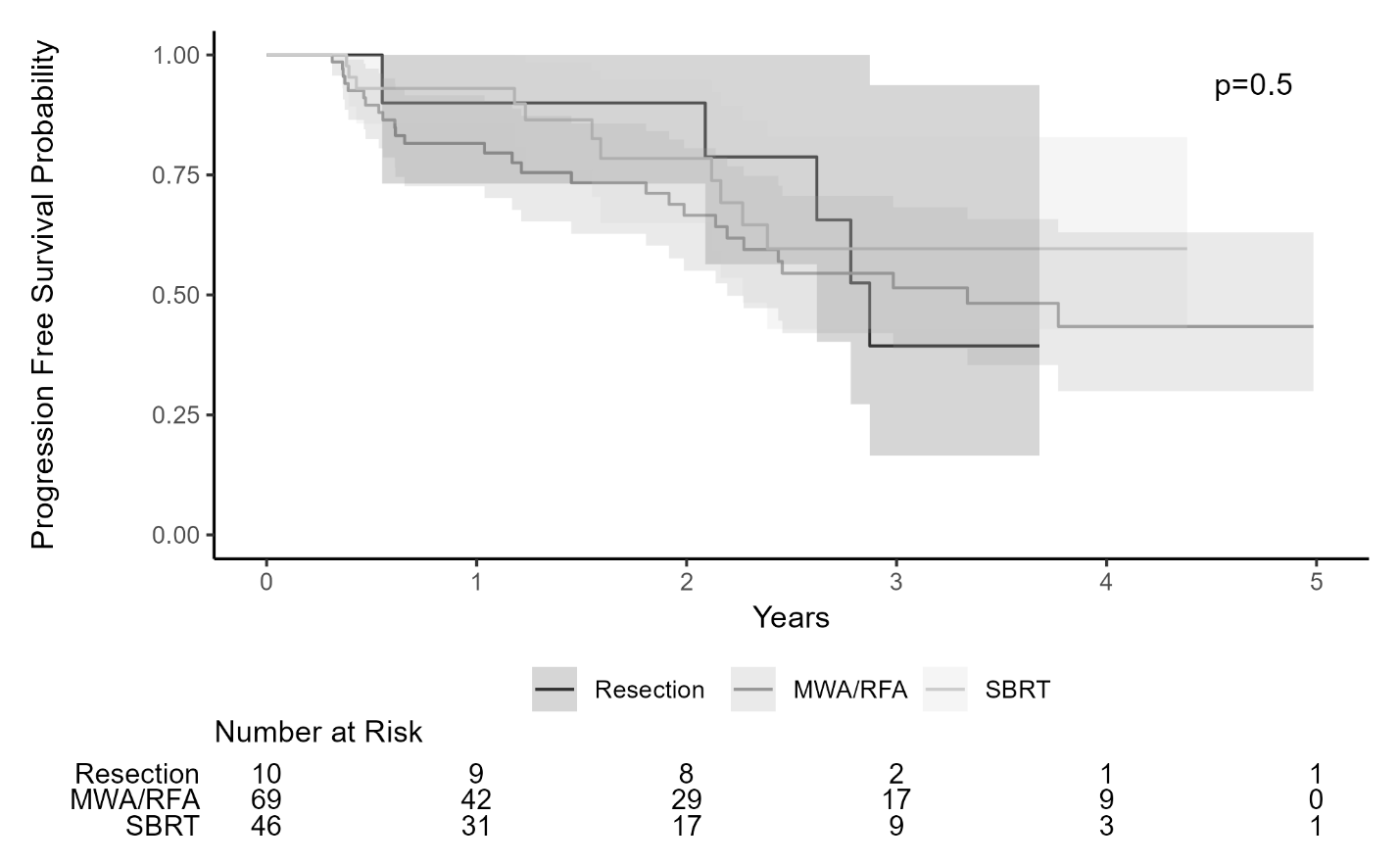

Supplement: Supplementary file 1 — Figures S1–S11: apt70438‐sup‐0001‐FigureS1‐S11.docx. [file APT-63-850-s001.docx]
